# Remedy for Hydrophobia

**Published:** 1850-02

**Authors:** 


*Remedy for Hydrophobia.*—M. ROCHER D'HÉRICOURT, who has lately returned from a journey in Abyssinia, has brought with him manuscripts of great literary value, and has collected all the facts calculated to throw light on geology, mineralogy, botany, and other branches of science. He has likewise brought with him numerous specimens of a plant, the root of which, reduced to powder, is a cure for hydrophobia, both in men and animals. Of its virtues M. d'Héricourt had practical proofs. Four dogs and a man having been bitten by a mad dog, they were, by the application of this remedy, cured of the hydrophobia which ensued; whilst the fourth dog (bitten at the same time, by the same animal), to which the remedy was not applied, perished in all the agony of that terrible disease. The virtue of the plant, and the manner of preparing it for use, were explained to the traveller by a potentate of the country, who assured him that it was there generally used, and never failed. The specimens brought over by M. d'Héricourt have been submitted to the Academy of Science of Paris, and a committee has been appointed to test their efficacy.—*Lancet.*

